# GENESIS: a French national resource to study the missing heritability of breast cancer

**DOI:** 10.1186/s12885-015-2028-9

**Published:** 2016-01-12

**Authors:** Olga M. Sinilnikova, Marie-Gabrielle Dondon, Séverine Eon-Marchais, Francesca Damiola, Laure Barjhoux, Morgane Marcou, Carole Verny-Pierre, Valérie Sornin, Lucie Toulemonde, Juana Beauvallet, Dorothée Le Gal, Noura Mebirouk, Muriel Belotti, Olivier Caron, Marion Gauthier-Villars, Isabelle Coupier, Bruno Buecher, Alain Lortholary, Catherine Dugast, Paul Gesta, Jean-Pierre Fricker, Catherine Noguès, Laurence Faivre, Elisabeth Luporsi, Pascaline Berthet, Capucine Delnatte, Valérie Bonadona, Christine M. Maugard, Pascal Pujol, Christine Lasset, Michel Longy, Yves-Jean Bignon, Claude Adenis, Laurence Venat-Bouvet, Liliane Demange, Hélène Dreyfus, Marc Frenay, Laurence Gladieff, Isabelle Mortemousque, Séverine Audebert-Bellanger, Florent Soubrier, Sophie Giraud, Sophie Lejeune-Dumoulin, Annie Chevrier, Jean-Marc Limacher, Jean Chiesa, Anne Fajac, Anne Floquet, François Eisinger, Julie Tinat, Chrystelle Colas, Sandra Fert-Ferrer, Clotilde Penet, Thierry Frebourg, Marie-Agnès Collonge-Rame, Emmanuelle Barouk-Simonet, Valérie Layet, Dominique Leroux, Odile Cohen-Haguenauer, Fabienne Prieur, Emmanuelle Mouret-Fourme, François Cornélis, Philippe Jonveaux, Odile Bera, Eve Cavaciuti, Anne Tardivon, Fabienne Lesueur, Sylvie Mazoyer, Dominique Stoppa-Lyonnet, Nadine Andrieu

**Affiliations:** Cancer Research Centre of Lyon, CNRS UMR5286, Inserm U1052, Université Claude Bernard Lyon 1, Centre Léon Bérard, Lyon, France; Unité Mixte de Génétique Constitutionnelle des Cancers Fréquents, Hospices Civils de Lyon, Centre Léon Bérard, Lyon, France; Inserm, U900 Paris, France; Institut Curie, Paris, France; PSL Research University, Paris, France; Mines ParisTech, Fontainebleau, France; Institut Curie, Service de Génétique, Paris, France; Institut de Cancérologie Gustave Roussy, Service d’Oncologie Génétique, Villejuif, France; Hôpital Arnaud de Villeneuve, CHU Montpellier, Service de Génétique médicale et Oncogénétique, Montpellier, France; ICM Val d’Aurel, Unité d’Oncogénétique, Montpellier, France; Centre Catherine de Sienne, Service d’Oncologie Médicale, Nantes, France; Centre Eugène-Marquis, Service de Génétique, Rennes, France; CH Georges Renon, Service Oncogénétique pour la consultation oncogénétique régionale Poitou-Charentes, Niort, France; Centre Paul Strauss, Unité d’Oncologie, Strasbourg, France; Institut Curie, Hôpital René Huguenin, Saint-Cloud, France; Hôpital d’Enfants, Service de Génétique Médicale, Dijon, France; Centre Georges François Leclerc, Oncogénétique, Dijon, France; ICL Alexis Vautrin, Unité d’Oncogénétique, Vandœuvre-lès-Nancy, France; Centre François Baclesse, Unité de pathologie gynécologique, Caen, France; Centre René Gauducheau, Unité d’Oncogénétique, Nantes Saint Herblain, France; Université Claude Bernard Lyon 1, Villeurbanne, France; CNRS UMR 5558, Lyon, France; Centre Léon Bérard, Unité de Prévention et Epidémiologie Génétique, Lyon, France; Hôpitaux Universitaires de Strasbourg, UF1422 Oncogénétique moléculaire, Laboratoire de diagnostic génétique, Strasbourg, France; Hôpitaux Universitaires de Strasbourg, UF6948 Oncogénétique, Service d’Hémato-Oncologie, Strasbourg, France; Inserm, U896, CRCM Val d’Aurel, Montpellier, France; Institut Bergonié, Bordeaux, France; Centre Jean-Perrin, Clermont-Ferrand, France; Centre Oscar-Lambret, Lille, France; Hôpital Universitaire Dupuytren, Service d’Oncologie Médicale, Limoges, France; Polyclinique Courlancy, Reims, France; Clinique Sainte Catherine, Avignon, France; CHU de Grenoble, Hôpital Couple-Enfant, Département de Génétique, Grenoble, France; Centre Antoine Lacassagne, Unité d’Oncogénétique, Nice, France; Institut Claudius Regaud – IUCT-Oncopole, Service d’Oncologie Médicale, Toulouse, France; Hôpital Bretonneau, Service de Génétique, Tours, France; CHU Brest, Hôpital Morvan, Département de génétique médicale en pédiatrie, Brest, France; Hôpital Tenon, Paris, France; Hôpital Edouard Herriot, Service de Génétique Moléculaire, Lyon, France; Hôpital Jeanne de Flandre, Service de génétique clinique Guy Fontaine, Lille, France; Hôpital Universitaire de Rouen, Département de Génétique, Rouen, France; Hôpital Pasteur, Service d’Onco-hématologie, Colmar, France; CHRU Hôpital Caremeau, Nîmes, France; Hôpital Tenon, Service d’Oncogénétique, Paris, France; IPC, Département d’Anticipation et de Suivi des Cancers, Marseille, France; Inserm, UMR 912, Marseille, France; Groupe Hospitalier Pitié-Salpêtrière, Département de Génétique, APHP, Paris, France; Centre Hospitalier de Chambéry, Chambéry, France; Institut Jean-Godinot, Reims, France; ICC Courlancy, Cs Oncogénétique, Reims, France; CHU Hôpital Saint-Jacques, Service Génétique et Biologie du Développement - Histologie, Besançon, France; Hôpital Flaubert, Le Havre, France; Hôpital Saint-Louis, Paris, France; CHU de Saint-Etienne, Hôpital Nord, Service de Génétique, Saint-Etienne, France; Hôpital Lariboisière, Centre Viggo-Petersen, Paris, France; CHU Hôpital de Brabois, Laboratoire de Génétique, Vandœuvre-lès-Nancy, France; CHU de Martinique, Unité d’Oncogénétique, Fort-de-France, France; Institut Curie, Département d’imagerie médicale, Paris, France; Inserm, U830, Paris, France; Université Paris-Descartes, Paris, France

## Abstract

**Background:**

Less than 20 % of familial breast cancer patients who undergo genetic testing for *BRCA1* and *BRCA2* carry a pathogenic mutation in one of these two genes. The GENESIS (GENE SISter) study was designed to identify new breast cancer susceptibility genes in women attending cancer genetics clinics and with no *BRCA1/2* mutation.

**Methods:**

The study involved the French national network of family cancer clinics. It was based on enrichment in genetic factors of the recruited population through case selection relying on familial criteria, but also on the consideration of environmental factors and endophenotypes like mammary density or tumor characteristics to assess potential genetic heterogeneity. One of the initial aims of GENESIS was to recruit affected sibpairs. Siblings were eligible when index cases and at least one affected sister were diagnosed with infiltrating mammary or ductal adenocarcinoma, with no *BRCA1/2* mutation. In addition, unrelated controls and unaffected sisters were recruited. The enrolment of patients, their relatives and their controls, the collection of the clinical, epidemiological, familial and biological data were centralized by a coordinating center.

**Results:**

Inclusion of participants started in February 2007 and ended in December 2013. A total of 1721 index cases, 826 affected sisters, 599 unaffected sisters and 1419 controls were included. 98 % of participants completed the epidemiological questionnaire, 97 % provided a blood sample, and 76 % were able to provide mammograms. Index cases were on average 59 years old at inclusion, were born in 1950, and were 49.7 years of age at breast cancer diagnosis. The mean age at diagnosis of affected sisters was slightly higher (51.4 years). The representativeness of the control group was verified.

**Conclusions:**

The size of the study, the availability of biological specimens and the clinical data collection together with the detailed and complete epidemiological questionnaire make this a unique national resource for investigation of the missing heritability of breast cancer, by taking into account environmental and life style factors and stratifying data on endophenotypes to decrease genetic heterogeneity.

## Background

Less than 20 % of women affected by breast cancer (BC) and qualified for *BRCA1/2* testing are carrying a deleterious (or pathological) mutation in one of these genes [1]. Mutations in other genes causing familial syndromes in which BC incidence is highly increased (*TP53, PTEN, STK11* and *CDH1*) are estimated to cause 5 % of the familial forms of BC and an additional 5 % is accounted for by moderate penetrance genes (i.e. associated with an odds ratio (OR) below 3), such as *ATM, CHEK2,* and the Fanconi anemia pathway genes (*BRIP1, PALB2, RAD51C, RAD51D* and *XRCC2*). Therefore, the majority of the familial forms of BC remain unexplained. Schematically, the studies performed to elucidate the missing heritability have either been on high-risk populations using mainly linkage analysis approaches to detect “major” genes or on the general population using association studies to detect “more” common genetic “variations”. Linkage analyses failed to identify new “major” loci [2] while genome-wide association studies performed on large case-control studies of BC have identified about 100 common BC susceptibility loci (single nucleotide polymorphisms or SNPs) to date (e.g. [3–13]). However, the effect sizes detected by these large-scale studies were small, for the vast majority, the associated OR rarely being greater than 1.20, and altogether may account for only 14 % of the missing heritability [14].

There is likely a genetic heterogeneity, with different types of predisposing situations observed among women at risk. These genetic “sub-entities” resulting from the combination of several factors may be associated with particular characteristics of the individual or of the tumor. For example, several SNPs identified by genome-wide association studies were shown to be associated with the estrogen receptor status of the breast tumor both in the general population [3, 4, 15–17] and the population of *BRCA2* mutation carriers [18–21].

Our proposal was to set up a study to investigate the missing heritability of BC in a high-risk population with unrelated controls for conducting association studies. The novelty of the GENESIS (GENE SISters) study is the recruitment of a study population enriched in susceptibility factors by case selection based on familial criteria, with consideration of environmental factors. Potential genetic heterogeneity was accounted for by stratifying the study sample on proxy such as particular individual epidemiological or clinical characteristics (mammary density, for example), or tumor characteristics.

The GENESIS study is an integrative genetic epidemiological project based on the involvement of all French family cancer clinic consultants who belong to the “Groupe Génétique et Cancer” (GGC) of Unicancer, the centralized enrolment of patients and collection of their clinical, epidemiological, familial and biological data by a coordinating center (CC) at the Institut Curie (Paris, France). Here we describe the design and logistics of the study and the available data. We also discuss the participation rates, the prevalence of the BC cases, and the representativeness of the participants and of the population of controls.

## Methods

### Eligible individuals

Index cases (and their affected sisters) were identified through the French family cancer clinics of the GGC (i.e. 42 centers) and were eligible when diagnosed with infiltrating mammary or ductal adenocarcinoma, were negative for *BRCA1* and *BRCA2* mutations, and had a sister with BC. The mutation screening strategy was similar for all the clinics. The full coding sequences and the exon-intron junctions of the *BRCA1* and *BRCA2* genes were screened for mutations, based on pre-screening (Denaturing High-Performance Liquid Chromatography (dHPLC), High-Resolution Melting (HRM) or Enhanced Mismatch Mutation Analysis (EMMA)) and sequencing. For a subset of the index cases; large rearrangements were screened by large cDNA sequencing, Multiplex Ligation-dependent Probe Amplification (MLPA), Quantitative Multiplex PCR of Short Fragments (QMPSF), Quantitative PCR (qPCR), Quantitative PCR High Resolution Melting (qPCR HRM), EMMA or dedicated array Comparative Genomic Hybridization (array CGH). Sisters with infiltrating mammary adenocarcinoma or *in situ *ductal carcinoma, regardless of their age at diagnosis, were eligible. If the index case had more than one affected sister, all were approached. Two types of controls were included: unrelated controls and unaffected sisters. The unrelated controls were selected among the unaffected friends and/or colleagues of the cases. The year of birth of controls was matched to that of the corresponding case (+/− 3 years). The parents, brothers and unaffected sisters were also contacted, when possible.

Geneticists of family cancer clinics identified index cases and invited them to participate in GENESIS by referring them to the CC. Each family cancer clinic invited index cases to participate in the study by letter (retrospective index cases with a molecular diagnosis performed between 2003 and 2007) or during consultations informing patients of their *BRCA1/2* negative results (prospective index cases). The CC organized the inclusion of index cases and of their relatives and unrelated controls. The index case then sent a response coupon to the CC to obtain the complete study file including a detailed information letter and a consent form to be completed. Subjects were included in the study when they sent back their signed consent, with the possibility of a telephone contact with a member of the CC team and/or a genetic consultation for additional information. The index case contacted her sisters (affected and unaffected), parents and brothers, and unrelated unaffected friends or colleagues and gave them an information letter and response coupon. After their agreement, the CC sent them the study file including the detailed information letter and the consent form. Again, relatives and unrelated subjects were included in the study when they sent back their signed consent. The CC organized the collection of blood samples from the index case and other participants by sending them a prescription for blood sampling, a letter for the medical analysis laboratory or the nurse who took the blood sample, and appropriate prepaid packaging for dispatch of the samples directly to the biological resource center at the Centre Léon Bérard (Lyon, France).

The study was examined by the appropriate committees: ethics (CCP Ile-de-France III, 3 October 2006, agreement no. 2373) and by the data protection agency (CNIL, 22 May 2006, agreement no. 1170775), all of which approved the study.

### Data collected

All patients (index cases and affected sisters) and controls completed a questionnaire on environmental, lifestyle and reproductive factors and family history of cancer. This self-report questionnaire contained detailed questions concerning demographic data, alcohol and tobacco consumption, pregnancies, breastfeeding, contraception, hormone replacement, physical activity, personal medical history and exposure to irradiation at work and for medical purposes and pedigree, with detailed information on medical history for each first- and second-degree relative.

Mammograms taken at the time of diagnosis or one to three years before diagnosis for the cases and most recent mammography for the controls and unaffected sisters at inclusion were collected. Craniocaudal and medio-lateral oblique views of both breasts were digitized. A VIDAR DiagnosticPro Advantage scanner, with a resolution of 570 dpi, was used to record the information required for quantitative calculations of mammary density. The images obtained were recorded and the identity of the participant was erased from images with Adobe® Photoshop® software. The incidence and date of the mammograms were recorded on the images.

Blood samples were collected from index cases, affected and unaffected sisters and controls. Viable lymphocytes from index cases were frozen if this had not already been done by the laboratory having carried out *BRCA1/2* analyses. Part of each blood sample was frozen, while the rest was processed in order to obtain plasma, serum, and lymphocyte pellets, all of which were then frozen. DNA was subsequently extracted using the AutopureLS Instrument (Qiagen).

No systematic collection of tumor specimens has been performed. However, pathology reports and information of sample storage conditions and location for mammary tumors have been collected and are being coded and computerized. This information will facilitate access to the tumor samples for specific projects to come.

### Study power

These data will be used to study the missing heritability of BC by taking into account environmental factors. The power of the study depends on the study design and strategy employed for detecting BC susceptibility alleles. For instance, SNP genotyping, mutation screening of candidate genes or whole exome sequencing may be undertaken in all subjects or specific subset of participants, since stratifying data on endophenotypes may help decreasing genetic heterogeneity. Interaction effects according to the genes under study may also be investigated. A “simple” power calculation showed that a genetic association with an amplitude of 3 (relative risk associated with a susceptibility genotype) sought by a candidate gene approach in a case-control study design can be identified with a power of 80 % (alpha = 0.05) for allelic frequencies greater than 0.5 % for a dominant inheritance and greater than 10 % for a recessive inheritance. The power will decrease with decreasing risk amplitude if the study eligibility criteria lead to an underrepresentation of high-risk families. *A priori* power calculations are challenging and will depend on the hypotheses and models used (single SNP analyses, gene pathway-based approaches, single environmental/lifestyle factor, exposure profiles with or without interaction…).

## Results

**Fig. 1:**
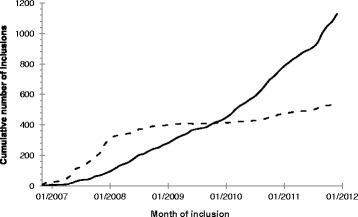
Cumulative number of index case inclusions over time. Legend:  Index cases with retrospective molecular diagnosis.  Index cases with prospective molecular diagnosis

Inclusion of participants started in February 2007 and ended in December 2013. Description of the GENESIS population is based on data available on 17 November 2014. Thus, the population may increase slightly when residual signed inform consents are received by the CC. Figure 1 shows the cumulative number of index cases over the time-course of inclusion, both for retrospective and prospective inclusions.

2543 patients qualified according to the study criteria were listed and invited to participate by 26 centers. 1669 women sent back a reply coupon to the CC for complete information about the study and 1315 agreed to participate (i.e. 52 % of patients invited). 539 additional cases, invited by 16 centers without being listed beforehand and meeting the inclusion criteria returned a reply coupon and 406 of them agreed to participate and were therefore included. A total of 1721 patients were included, 1483 of whom had at least one living sister affected by BC and potentially able to participate in the study, and 238 of whom had no living affected sister. Since the participation rate was not very high, the representativeness of the included cases could be questioned. Birth year, age at diagnosis and year of diagnosis were compared in the eligible population (631 index cases) of the Institut Curie clinic where the information was available. The characteristics of eligible index cases and the characteristics of index cases included were similar, i.e. 1948, 50 years and 1998 on average for birth year, age at diagnosis and year of diagnosis, respectively. Thus, we can be confident that the population of index cases is representative of the targeted population.

98 % of index cases completed the epidemiological questionnaire and provided a blood sample; 68 % were able to provide mammograms. The information on mammograms was extracted from the epidemiological questionnaire. The collection of mammograms is still on-going since many are kept in the care centers where the women were treated for their cancer (cf. Table 1). Table 2 shows that all index cases included were on average 59 years old at inclusion (minimum (min): 31 years old; maximum (max): 90 years old), were born in 1950 (min: 1918; max: 1977) and were 49.7 years old at the BC diagnosis (min: 20 years old; max: 80 years old). The mean interval between the date of diagnosis and the date of inclusion was 9.3 years (min: 0 year; max: 48 years).

**Table 1:** Available data in GENESIS per type of participant

Type of participant	Signed consent form	Completed questionnaire	Blood sample	At least one mammogram performed^a^	At least one mammogram collected
	N	N (%)	N (%)	N	N (%)
Index case	1721	1682 (98 %)	1695 (98 %)	1721	1169 (68 %)
Affected sister	826	807 (98 %)	805 (97 %)	826	546 (66 %)
Unaffected sister	599	592 (99 %)	582 (97 %)	589	493 (84 %)
Control	1419	1411 (99 %)	1360 (96 %)	1322	1201 (91 %)
Total	4565	4492 (98 %)	4442 (97 %)	4458	3409 (76 %)

N, number; %, percentage, ^a^based on questionnaire information for unaffected women

**Table 2:** Characteristics of the GENESIS population according to the type of participant

Type of participant		Year of birth	Age at breast cancer diagnosis (years)	Age at inclusion (years)	Interval between diagnosis and inclusion			
						By class		
						≤4 years	5-9 years	≥10 years
	N	mean (SD) [min, max]	mean (SD) [min, max]	mean (SD) [min, max]	mean (SD)	N (%)	N (%)	N (%)
Index case	1682	1950 (9.3) [1918, 1977]	49.7 (9.3) [20, 80]	59.0 (9.3) [31, 90]	9.3 (7.4)	559 (33 %)	480 (29 %)	643 (38 %)
Affected sister	807	1949 (9.0) [1926, 1978]	51.4 (8.7) [27, 77]	59.5 (8.8) [30, 83]	8.1 (6.4)	297 (37 %)	249 (31 %)	258 (32 %)
Unaffected sister	592	1952 (9.3) [1926, 1978]		57.0 (9.2) [30, 84]				
Control	1411	1953 (10.0) [1926, 1991]		55.7 (9.9) [19, 83]				

N, number; SD, standard deviation; min, minimum; max, maximum; %, percentage

This interval has an effect on the familial phenotype of the index cases. 32 % of the index case families had 3 or more cases of BC when the interval was greater than 10 years; this percentage decreases to 26 % when the interval was less than 10 years. This difference is mostly due to a longer survey of the families. When the follow-up of the family members was censured at the year of diagnosis of the index case, the percentage of index case families with 3 or more BC cases was 24 % when the interval was greater than 10 years and 23 % when it was less than 10 years. Thus, ascertainment by familial phenotype criteria appears constant over time.

Of the 5095 sisters and controls invited by the index cases and identified through the list of invitations sent back by the index case, 2518 women agreed to participate, i.e. 685 sisters with BC (57 %), 541 unaffected sisters (54 %) and 1292 controls (45 %). An extra 141 affected sisters, 58 unaffected sisters and 127 controls, not previously listed in the index cases’ invitation list, agreed to participate. Thus a total of 826 affected sisters, 599 unaffected sisters and 1419 controls were included.

98 % of the affected sisters and 99 % of the unaffected sisters and controls completed the epidemiological questionnaire, and 97 % of the sisters and 96 % of the controls provided a blood sample. 66 % of the affected sisters provided mammograms, a percentage similar to that of the index cases, again because many of the mammograms are kept in the care centers where the sisters with BC were treated for cancer. The percentage was higher for the unaffected sisters and the controls: 84 % and 91 %, respectively (cf. Table 1). The mean ages at diagnosis and at inclusion of the affected sisters were slightly higher than those of the index cases, i.e. 51.4 and 59.5 years, respectively, with therefore a slight decrease in the interval between diagnosis and inclusion (8.1 years vs 9.3 years). On average, the unaffected sisters were two years younger than the index cases and the controls three years younger (cf. Table 2).

Our target of recruiting 1000 sibpairs, based on power calculations, was not reached. Among the 1483 index cases with at least one sister alive and with BC, 696 had at least one affected sister included (47 %). A total of 788 affected sisters agreed to participate. The size of the sibships with participating affected sisters varied from 2 to 5 (cf. Table 3). Of the 696 sibships with at least two affected sisters included, 678 completed the epidemiological questionnaires, 669 provided blood samples and 362 provided mammograms. The absolute mean difference in the ages at diagnosis within sibpairs was 5.7 years (Standard Deviation (SD): 8.2 years).

**Table 3:** Available data on sibships with affected sisters

Number of sisters with breast cancer per sibship	Number of sibships with			
	Signed consent forms	Completed questionnaires	Blood samples	Mammograms collected
at least 2^a^	696	678	669	362
exactly 2^a^	616	600	592	324
exactly 3^a^	69	67	67	34
exactly 4^a^	10	10	9	4
exactly 5^a^	1	1	1	0

^a^Whether the women are index cases or affected sisters

Since the controls were supposed to be either a friend or a colleague of an index case, the representativeness of this group is questionable in terms of family history of cancer. Indeed, friends or colleagues may have participated because they have a “strong” family history of cancer. This bias might lead to an underestimate of the effect of genetic factors. Therefore, we analyzed the pedigree of the 1411 unrelated controls by comparing the cancer incidences in these pedigrees (first degree of relationship) to the national incidences using SAS 9.3 software (SAS Institute, Cary, NC) and the estimated national cancer incidences from 1975 to 2005 [22]. We observed a slight increased incidence of cancer within control families for all sites (Standardized Incidence Ratio (SIR): 1.11 95%CI: 1.02–1.22), for breast cancer (SIR: 1.21 95%CI: 1.04–1.39) and for ovarian cancer (SIR: 1.16 95%CI: 0.73–1.76) and this should be taken into account in the further analyses.

## Discussion

The GENESIS study is a unique national resource to study the missing heritability of BC. This is a large study including over 6000 participants, and the biological, clinical and epidemiological data collected are detailed and complete. The rate of agreement to participate was moderate for each participant category, around 50 %. However, the constraint of asking the index cases to contact the other participants impaired assessment of the true agreement rates as for these people we relied on a list completed by the index cases. Indeed, people not listed beforehand were included (more than 11 % of the inclusions). Even though we have full confidence in the representativeness of the population of the included index cases and controls, it may be more effective in future studies to contact relatives and other participants directly.

Even though part of the study was prospective, most index cases and their affected sisters were prevalent cases (only 5.5 % were included in the calendar year of their BC diagnosis or in the following year). This has to be taken into account when seeking for BC susceptibility genes in order to avoid false conclusion. For instance, identified genes could be in fact involved in survival; conversely, one could miss BC susceptibility genes that are also involved in poor prognosis. Hence, for future studies using this resource, a comparison between pseudo-incident cases (interval between diagnosis and interview less than 5 years, for example) and prevalent cases will be useful.

The unrelated controls were matched to the corresponding index cases on the basis of the year of birth (+/− 3 years) to simplify the index cases’ task of inviting controls. However, because the large majority of index cases were included prospectively, their invited controls had survived without BC years after the index cases’ year of diagnosis. This may therefore result in bias toward the alternative even after censuring controls at the index cases’ age at diagnosis. To avoid such bias, depending on the question under study, the controls could be matched either to the index case’s year of birth or age at BC diagnosis. The latter option should be avoided when a cohort effect is observed for the variables under study.

The eligibility criteria of GENESIS did not exclude patients carrying a mutation in “clinically actionable” susceptibility genes other than *BRCA1* and *BRCA2*. In France, *PALB2* testing was introduced in the clinical practices in July 2015; therefore *PALB2* status in GENESIS index cases was not available at the time of inclusion. Subsequently, a case-control study performed in French BC families and including the first 40 % of the recruited GENESIS index cases has shown that *PALB2* truncating mutation are found in 0.36 % of the familial cases [23]. Whole-exome and whole-genome sequencing projects are also ongoing on a subset of the GENESIS population, as well as targeted sequencing of a panel of high- to moderate-risk genes for more than 2000 GENESIS participants. Genes under investigation includes, among others, *ATM, CHEK2, RAD51* paralogs and those included in panels used by the French diagnosis laboratories for research purpose (Lesueur et al. personal communication). Subjects carrying a mutation in one of these genes will be therefore identifiable for subsequent studies.

The low number of large families for which nearly all first-degree relatives have been recruited might be a limitation for powerful co-segregation analyses. However, in order to validate new potential BC susceptibility genes, it will be possible to use additional large sample set thanks to the GGC families’ recruitment or through participation to other international high-risk family studies.

## Conclusions

The identification of new BC susceptibility genes will clearly have implications for the management of women at risk. It will enable adaptation of the follow-up of these women according to risk assessments based on new tests. If the risk is considered high, early and regular MRI-based screening could be offered.

The identification of new genes should improve our understanding of the origin of a proportion of BC sporadic cases and should make it possible to optimize the management of these cases’ relatives.

Finally, an understanding of the biological functions of these genes and their interactions with environmental factors may reveal new possibilities for the prevention and treatment of BC, the incidence of which is continuing to increase in Western populations.

The GENESIS study will be an asset for ongoing molecular studies aiming at identifying new BC susceptibility genes, and such studies using this resource have already started (e.g. [23–25]). GENESIS resource also contributes to international consortia like COMPLEXO (COMPLexity of EXOme) [26].

### Special dedication

This article is dedicated to the memory of Olga M. Sinilnikova who died prematurely on June 30, 2014. Olga participated decisively in structuring research around hereditary predisposition to BC and in leading the GENESIS study.

## Abbreviations

BC: Breast cancer
CC: Coordinating Center
GGC: Groupe Génétique et Cancer
min: Minimum
max: Maximum
OR: Odds ratio
SD: Standard deviation
SIR: Standardized incidence ratio
SNP: Single nucleotide polymorphism
dHPLC: Denaturing high-performance liquid chromatography
HRM: High-resolution melting
EMMA: Enhanced mismatch mutation analysis
MLPA: Multiplex ligation-dependent probe amplification
QMPSF: Quantitative multiplex PCR of short fragments
qPCR: Quantitative PCR
qPCR HRM: Quantitative PCR High Resolution Melting
array CGH: Array comparative genomic hybridization
